# The influence of positive mental health and work stress on resilience among mental health nurses: a cross-sectional study

**DOI:** 10.1186/s12909-025-08249-6

**Published:** 2025-12-17

**Authors:** Adnan Innab, Atallah Alenezi, Sahar Al-Ghareeb, Mona Mostafa

**Affiliations:** 1 Nursing Administration and Education Department, College of Nursing, King Saud University, Riyadh, 12372 Saudi Arabia; 2 College of Nursing, Imam Mohammad Ibn Saud Islamic University (IMSIU), Riyadh, Saudi Arabia; 3 Fundamentals of Nursing Department, College of Nursing, Imam Abdulrahman Bin Faisal University, Dammam, Saudi Arabia; 4 Department of Nursing Sciences, College of Applied Medical Sciences, Shaqra University, Shaqra, Reyad, Saudi Arabia

**Keywords:** Nurses, Resilience, Workplace stress, Mental health, Social support, Emotion coping, Situation coping

## Abstract

**Background:**

Mental health nursing is classified as a high-risk profession, as these nurses face high stress levels, which have detrimental effects on nurses’ mental, emotional, and physical well-being.

**Aim:**

This study assessed the influence of positive mental health and work stress on resilience among mental health nurses.

**Methods:**

A cross-sectional, correlational, descriptive design was applied. Convenience sampling was used to collect data (from January to December 2024) using three instruments: the Positive Mental Health Scale, Workplace Stress Scale, and Resilience Appraisal Scale.

**Results:**

Participants (*N* = 300) reported moderate stress levels (M = 24.8, SD ± 4.56), with major concerns being a lack of adequate recognition for good performance and the belief that their job negatively affects their physical or emotional well-being. The total mental health score was 2.82 (SD ± 0.47), indicating a perception of good positive mental health among participants. Participants reported moderate social support (M = 3.40, SD ± 0.66), emotion coping (M = 3.26, SD ± 0.67), and situation coping (M = 3.22, SD ± 0.68). The mean resilience score was 3.29 (SD ± 0.54), suggesting an ability to effectively manage some situations but not others. Work stress was correlated with lower levels of positive mental health, social support, situation coping, and resilience. Positive mental health (*p* < 0.001) and work stress (*p* = 0.015) were significant predictors of resilience, accounting for 24.6% of the variance.

**Conclusions:**

Effective resilience interventions are crucial to reduce work-related stress and improve nurses’ performance. Creating supportive work environments that foster collaboration and empower nurses to alleviate work-related stress, enabling them to deliver high-quality care.

**Supplementary Information:**

The online version contains supplementary material available at 10.1186/s12909-025-08249-6.

## Background

Nurses are the backbone of healthcare, and their work is essential to global health outcomes. Investing in their mental health and well-being is a global health imperative. Mental health nursing (MHN) is a distinct field marked by increasing research on occupational stress and general well-being [1]. Mental health nursing is a demanding profession; these nurses experience considerable stress, and their careers are classified as high-risk [2]. Occupational stress negatively affects their mental health, job satisfaction, and well-being [3].

Mental health nurses encounter multiple stressors, including workplace violence, excessive workloads, inadequate recognition from staff, poor relationships with patients, and strained professional interactions [4, 5]. Psychiatric nursing is inherently stressful because of the emotional demands of caring for patients with complex mental health conditions [6, 7]. According to a systematic review and thematic synthesis, workplace stress among psychiatric nurses is associated with long shifts, emotionally intense interactions, and exposure to violence in the workplace [8]. In Saudi Arabia, mental health nurses face additional stressors, including challenges linked to cultural norms, societal stigmas surrounding mental illness, and limited healthcare resources [9]. Additionally, they operate in a demanding and rapidly changing environment, in which emotional resilience is essential, and high nurse-to-patient ratios are prevalent. Extended exposure to such stress can lead to burnout, reduced job satisfaction, and diminished patient care quality [10, 11].

According to the World Health Organization, positive mental health enables individuals to effectively manage their stress, understand their potential, and have a sense of belonging [12]. Positive mental health is a state that enables individuals to cope effectively with work stressors, overcome challenges, work productively, and make a valuable contribution to the community [12]. Positive mental health is not only the absence of mental illness but the presence of a positive mental status, characterized by resilience and positive emotions. Research indicates the importance of positive mental health in improving mental status and enhancing resilience [6–8]. The discussion on positive mental health highlights the importance of implementing workplace strategies that enhance mental well-being and foster resilience. These strategies play a crucial role in improving adaptability, reducing stress, and promoting resilience for both individuals and organizations [13]. Research has demonstrated a significant positive correlation between positive mental health and resilience. This suggests that positive mental health plays a significant role in enhancing resilience in stressful work environments [14].

Resilience is a dynamic process that involves adaptability at the psychological, emotional, and behavioral levels. An integrative review of 15 studies conducted internationally indicated that mental health nurses exhibited moderate to high levels of resilience, which were inversely associated with mental distress [15]. Several international studies have addressed the key role of resilience in improving stress management, mental health, and long-term professional performance for healthcare professionals [16–19].

Previous research has emphasized the importance of resilience in mental health professionals. For example, a comprehensive review found that resilience programs improved personal awareness, coping strategies, self-reliance, and career-related relationships. In mental health settings, resilience involves emotional, cognitive, and behavioral self-regulation, drawing on both internal resources (e.g., positive emotion regulation and cognitive evaluation) and external resources (e.g., support) [20]. These resources may help nurses adapt and regain full functional capacity after encountering challenges in the workplace. Other researchers highlight the significant role of resilience in mitigating the effects of work-related stress, anxiety, and mental distress among mental health nurses [21].

Mental health issues in nurses can adversely affect their physical well-being, lower their quality of life, compromise service delivery, endanger patient safety, increase turnover rates, and exacerbate workforce shortages [6]. It is crucial to have a proper understanding of how mental health nurses cope with work stress, enhance their positive mental health, and develop resilience. The Conservation of Resources (COR) theory provides a solid basis for this research, as it emphasizes the importance of acquiring, safeguarding, and replenishing personal resources to manage stress and foster resilience effectively. Thus, COR theory provides a pertinent framework for examining the relationships between work-related stress, positive mental health, and resilience [22].

Prior studies have typically examined work stress and resilience separately, indicating that mental health nurses in Saudi Arabia experience moderate stress levels mainly due to excessive workloads, while also exhibiting high resilience. However, the connection between these factors and positive mental health has seldom been thoroughly investigated [10]. One study investigated the effects of mental well-being and stress on resilience, but did not focus specifically on mental health nurses [11]. Furthermore, although some research in Saudi Arabia reveals a connection between resilience and work-related stress in mental health settings [10], the impact of positive mental health on work-related stress and the resilience of psychiatric nurses in Saudi Arabia remains underexplored. Despite previous research, significant gaps remain in understanding the association between positive mental health, work-related stress, and resilience among mental health nurses.

By combining insights from both Saudi and global research, a fresh perspective is starting to emerge. This study notably introduces positive mental health as a variable, representing a significant shift from previous research. It enables a deeper examination of how positive mental health strategies influence the impact of workplace stress [23, 24]. This research exposes the substantial impact of positive mental health and work-related stress on the resilience of mental health nurses, thereby contributing to organizational advancement. It holds relevance not only within the context of Saudi Arabia but also across the global landscape of international mental health nursing [10, 11, 23, 24].

### Theoretical framework

This study was guided by the conservation of resources (COR) theory, developed by Hobfoll (1989). This theory posits that individuals are inherently motivated to acquire, retain, and safeguard resources—categorized as objects, conditions, personal characteristics, and energy—and that the perceived or actual loss of these resources constitutes a primary source of stress, exceeding the impact of never having them [22].

The (COR) theory explains how individuals cope with stress by emphasizing the importance of safeguarding and enhancing their resources. In this context, work-related stress is viewed as a factor that may lead to the depletion of vital resources such as energy, time, or support. Positive mental health is perceived as a crucial resource that enables individuals to remain resilient and recover from challenges. Resilience refers to the capacity to recover and persist in the face of stress, which the COR theory connects to effective resource management. The theory informed the research by proposing that individuals with greater positive mental health (i.e., more resources) are better equipped to cope with work stress (i.e., resource depletion), thereby enhancing their resilience. This indicates that the key concepts of the study were founded on the dynamics of resource acquisition, loss, and protection based on COR principles [22, 25].

An understanding of resource management dynamics, as delineated by COR theory, enables mental health practitioners, organizations, and individuals to implement targeted strategies to enhance coping mechanisms and support resilience, ultimately promoting adaptive stress responses [25].

### Purpose statement

This study assessed the influence of positive mental health and work stress on resilience. The specific aims were to (1) assess the level of work stress, positive mental health, and resilience; (2) examine the relationships between age, positive mental health, work stress, and resilience; (3) examine the mean differences in positive mental health, work stress, social support, emotional coping, situational coping, and resilience according to demographic characteristics, and (4) assess the influence of positive mental health and work stress on resilience.

## Methods

### Study design

The study utilized a cross-sectional, correlational, descriptive design and followed the reporting guidelines outlined in the Strengthening the Reporting of Observational Studies in Epidemiology (STROBE) statement.

### Sample and setting

This study was carried out at a governmental psychiatric hospital in Riyadh, Saudi Arabia. It is one of Saudi Arabia’s leading facilities specializing in psychiatric and addiction treatment. The hospital offers free clinical, preventive, and rehabilitative services, has a capacity of more than 500 beds, and delivers over 1.4 million medical and supportive services annually. It provides a wide range of care, including emergency, inpatient, outpatient, virtual consultations, home follow-ups, laboratory, and specialized clinics for children. Beyond treatment, the facility emphasizes psychosocial support, rehabilitation, volunteer engagement, and academic collaborations. It offers programs that empower patients, enhance recovery, and promote reintegration into society. These initiatives make the hospital a model of holistic, patient-centered mental health care in the Kingdom.

The inclusion criteria were: (1) being a registered nurse, (2) working in a mental health hospital or department, and (3) having at least two years of experience. Exclusion criteria included individuals who declined to participate, those absent due to illness or personal leave, and nursing interns.

G-Power (version 3.1.9) determined that a minimum of 92 participants was sufficient (power = 0.8, effect size = 0.15, α = 0.05). A total of 310 licensed mental health nurses volunteered; 10 incomplete surveys were excluded, resulting in 300 completed surveys.

Although the minimum required sample size was smaller, a total of 300 participants were recruited to increase statistical power, ensure adequate representation across demographic subgroups, and improve the precision and reliability of the analyses.

### Data collection

The study was conducted at a single, specialized mental health hospital in Saudi Arabia with clinics for mental health and addiction-related conditions. This facility offers free treatment services, along with preventive and rehabilitation programs. A convenience sampling approach was used to recruit participants. While this method facilitated timely recruitment, it may introduce selection bias and limit generalizability; this limitation was considered when interpreting the findings.

Following formal approval from the relevant authorities, participants received a link to a Google Forms survey via email. To maintain confidentiality, no identifiers was collected. To prevent duplicate submissions, the “limit to one response” option was activated in Google Forms. In addition, incomplete responses were excluded during data management. Data were collected from January to December 2024, and responses were subsequently downloaded and analyzed. The extended period was necessary to accommodate nurses’ varying work schedules, periods of leave, and workload demands, which affected their availability to participate. Spreading data collection over 12 months ensured adequate response rate.

### Measurements

The questionnaire included three instruments: the Positive Mental Health (PMH) Scale, the Workplace Stress Scale (WSS-8), and the Resilience Appraisal Scale (RAS). Demographic information, including age, gender, educational level, marital status, department, and years of experience, was also collected.

### PMH scale

The PMH Scale is a unidimensional scale that assesses specific aspects of participants’ mental health [i.e., well-being, life satisfaction, and overall mental health, rather than focusing specifically on mental illness] [26]. It consists of nine items measured on a 4-point Likert scale, ranging from 1 (strongly disagree) to 4 (strongly agree). The total score ranges from 0 to 36, with higher scores indicating greater positive mental health [26]. The psychometric properties of the scale have been tested in different languages and cultures and have demonstrated reliability and validity [26, 27]. In a validation study conducted on a Palestinian sample [28], the PMH scale demonstrated excellent internal consistency (α = 0.91). In a recent study Saudi Arabia [27], the scale also demonstrated high internal consistency (0.86). In this study, Cronbach’s alpha was 0.77, indicating adequate internal consistency and reliability.

### WSS-8

The WSS-8 is used to evaluate several factors that contribute to workplace stress, helping organizations identify stressors that impact employee well-being and productivity [29]. It consists of eight items covering common workplace-related stressors, allowing for efficient assessment. Score interpretation offers valuable insights into an individual’s stress levels and potential areas of concern. Responses are evaluated on a 5-point Likert scale, ranging from 1 (never) to 5 (very often), with higher scores indicating a worse condition. Scores are interpreted as follows: a total score below 20 is considered “fairly low,” a total score of 21 to 25 is considered “moderate stress,” a total score of 26 to 30 is considered “severe stress,” and a total score of 31 to 40 indicates that the stress level is “potentially dangerous.” Although the WSS-8 has been applied in studies of healthcare professionals in Saudi Arabia, no published validation has examined its internal consistency or construct validity in a Saudi sample. In this study, Cronbach’s alpha for the scale was 0.71.

### RAS

The RAS is used to assess how individuals view their own resilience and coping capabilities, which is crucial for mental health evaluations and interventions [30]. The scale consists of 12 items divided into three subscales (Social Support, Emotion Coping, and Situation Coping) that measure psychological resilience. Responses were assessed using a 5-point Likert scale, ranging from 1 (strongly disagree) to 5 (strongly agree). The total score is obtained by summing the individual item scores and ranges from 12 to 60. A higher total score reflects a robust perception of an individual’s resilience, implying that the individual believes they are well-prepared to cope successfully with stressors and difficulties. There is currently no published study reporting the internal consistency reliability of the Resilience Appraisal Scale (RAS-12) in an Arabic-speaking population, including Saudi Arabia. In this study, Cronbach’s alpha for the scale was 0.769, indicating adequate internal consistency reliability.

### Data analysis

The Statistical Package for the Social Sciences (SPSS) version 30 was used for data processing and statistical analysis. Descriptive statistics (frequency, mean, standard deviation, and percentage) were used to describe the demographic characteristics and instrument item analysis. Independent-samples t-tests and one-way analysis of variance (ANOVA) were used to assess differences in participants’ positive mental health, work stress, social support, emotional coping, situational coping, and resilience based on gender, education level, and years of experience. The assumptions of t-test and ANOVA were tested and met. Normality was confirmed through Q–Q plots and Shapiro–Wilk tests, and no significant outliers were detected. Homogeneity of variances was supported by Levene’s test (*p* > 0.05). These results indicate that the data satisfied the required conditions for ANOVA.

Pearson’s product-moment correlation was used to determine the relationship between age, positive mental health, work stress, and resilience. Multiple linear regression was used to assess the influence of positive mental health and work stress on resilience. All regression assumptions were met: residual plots confirmed linearity and homoscedasticity; the Durbin–Watson statistic (1.5–2.5) indicated independence of observations. The variance inflation factor values below 5 ruled out multicollinearity. The Q–Q plots showed normality; and Cook’s distance values below 1 confirmed no influential outliers.

### Ethical considerations

The study was approved by the Institutional Review Board of Imam Mohammed Ibn Saud Islamic University [ERC-SU-S-202400047]. The recruitment statement included information on the study’s purpose and potential risks and benefits, and it informed participants that participation was voluntary. Participants were asked to check a box indicating their agreement to participate. Participants remained anonymous, and data were reported in aggregated form. The data will be retained securely for 5-years after publication in compliance with institutional guidelines.

## Results

### Sample characteristics

Table 1 displays the demographic characteristics of the participants. Of the 310 individuals who completed the questionnaire, 10 were excluded because they provided incomplete responses, resulting in a final sample size of 300 participants. The response rate was 96.77. The mean age was 34 years (SD ± 8.89), and most participants were female (71%) and held a BSN degree or higher (51%). Nearly half of the participants had less than 10 years of experience (47.7%), and most were employed in acute care settings (68.7%).

**Table 1:** Participants’ demographic characteristics (*N* = 300)

Variable (Range)	*n* (%) or M (SD)
Age (21–66)	34.05 (8.89)
Gender	
Male	87 (29%)
Female	213 (71%)
Marital status	
Single	74 (24.7%)
Married	179 (59.7%)
Divorced	38 (12.7)
Widow	9 (3.0%)
Level of education	
Diploma	147 (49%)
BSN or higher	153 (51%)
Length of experience (years)	
< 10 years of experience	143 (47.7%)
11–20 years of experience	108 (36%)
> 30 years of experience	49 (16.3%)
Department type	
Acute care department	206 (68.7%)
Emergency	18 (6%)
Outpatient	76 (25.3%)

### Descriptive statistics of positive mental Health, Resilience, and stress

Table 2 presents the item analysis of work stress, positive mental health, and resilience. Participants reported moderate stress levels (M = 24.8, SD ± 4.56). However, the average work stress was nearly in the severe range. The item analysis revealed that the participants shared certain factors, including a lack of adequate recognition for good performance (M = 3.25, SD ± 0.97) and the belief that their job negatively affects their physical or emotional well-being (M = 3.17, SD ± 1.01). They reported often feeling overwhelmed by their workload, struggling to communicate their opinions to superiors, experiencing conflict between their work and personal life, and being unable to fully leverage their skills in the workplace. The item indicating that nurses adequately control their work duties showed the lowest mean agreement (M = 3.02, SD ± 1.01).

Regarding positive mental health, participants reported that they are generally confident (M = 2.93, SD ± 0.83) and calm (M = 2.90, SD ± 0.76). However, the items covering good physical and emotional conditions showed the lowest agreement (M = 2.72, SD ± 0.79) and being in good spirits (M = 2.73, SD ± 0.80). The total mean score for mental health was 2.82 (SD ± 0.47), indicating that in general, participants perceived their mental health as stable (Table 2).

Table 2 also presents the item and subscale analysis of resilience. The results revealed that participants had moderate social support (M = 3.40, SD ± 0.66), moderate emotion coping (M = 3.26, SD ± 0.67), and moderate situation coping (M = 3.22, SD ± 0.68). Agreement was notably low for specific situational coping items, such as “When faced with a problem, I can usually find a solution” (M = 3.09, SD ± 1.14), and “I can generally solve problems that occur” (M = 3.19, SD ± 1.4). The average resilience score was 3.29 (SD ± 0.54), suggesting that while participants can manage certain situations effectively, they may encounter challenges in others.

**Table 2:** Descriptive statistics of work stress, positive mental health, and resilience

Item/Scale Analysis	Mean	SD
Work Stress	24.8	4.56
Conditions at work are unpleasant or sometimes even unsafe.	3.07	0.984
I feel that my job is negatively affecting my physical or emotional well-being.	3.17	1.01
I have too much work to do and/or too many unreasonable deadlines.	3.11	1.04
I find it difficult to express my opinions or feelings about my job conditions to my superiors.	3.06	0.985
I feel that job pressures interfere with my family or personal life.	3.08	1.08
I feel that I have inadequate control or input over my work duties.	3.02	1.01
I receive inadequate recognition or rewards for good performance.	3.25	0.972
I am unable to fully utilize my skills and talents at work.	3.03	1.00
Positive Mental Health	2.82	0.47
I am often carefree and in good spirits.	2.73	0.80
I enjoy my life.	2.85	0.756
All in all, I am satisfied with my life.	2.84	0.843
In general, I am confident.	2.93	0.839
I manage well to fulfill my needs.	2.77	0.839
I am in good physical and emotional condition.	2.72	0.794
I feel that I am actually well-equipped to deal with life and its difficulties.	2.79	0.771
Much of what I do brings me joy.	2.84	0.755
I am a calm, balanced human being.	2.90	0.768
Resilience	3.29	0.547
Social Support	3.40	0.666
If I were to have problems, I have people I could turn to.	3.39	1.02
My family and friends are very supportive of me.	3.44	1.01
If I were in trouble, I know of others who would be able to help me.	3.37	0.922
I could find family and friends who listened to me if I needed them to.	3.40	1.03
Emotion Coping	3.26	0.673
In difficult situations, I can manage my emotions.	3.14	0.986
I can put up with my negative emotions.	3.43	0.991
I can control my emotions.	3.19	1.120
I can handle my emotions.	3.29	1.048
Situation Coping	3.23	0.689
When faced with a problem, I can usually find a solution.	3.09	1.144
I can generally solve problems that occur.	3.19	1.044
I can usually find a way of overcoming problems.	3.36	1.017
If faced with a setback, I could probably find a way around the problem.	3.25	1.003

### The relationship between age, positive mental health, work stress, and resilience

The relationship between age, positive mental health, work stress, and resilience is presented in Table 3. Age was negatively correlated with positive mental health (*r* = − 0.151, *p* < 0.01), social support (*r* = − 0.174, *p* < 0.01), emotion coping (*r* = − 0.183, *p* < 0.01), situation coping (*r* = − 0.116, *p* < 0.05), and resilience (*r* = − 0.194, *p* < 0.01). This indicates that older participants had lower positive mental health, emotion coping, situation coping, and resilience. Positive mental health was positively correlated with social support (*r* = 0.417, *p* < 0.01), emotion coping (*r* = 0.383, *p* < 0.01), situation coping (*r* = 0.378, *p* < 0.01), and resilience (*r* = 0.485, *p* < 0.01). Work stress was negatively correlated with positive mental health (*r* = − 0.180, *p* < 0.05), social support (*r* = − 0.176, *p* < 0.01), situation coping (*r* = − 0.226, *p* < 0.01), and resilience (*r* = − 0.209, *p* < 0.01). This suggests that nurses with higher work stress have lower positive mental health, social support, situation coping, and resilience.

**Table 3:** Association between Age, positive mental Health, work Stress, and resilience (*N* = 300)

Variables	1	2	3	4	5	6	7
1. Age	1						
2. Positive Mental Health	−0.151**	1					
3. Work Stress	0.008	−0.180**	1				
4. Social Support	−0.174**	0.417**	−0.176**	1			
5. Emotion Coping	−0.183**	0.383**	−0.103	0.472**	1		
6. Situation Coping	−0.116*	0.378**	−0.226**	0.455**	0.519**	1	
7. Resilience	−0.194**	0.485**	−0.208**	0.790**	0.820**	0.817**	1

Pearson’s correlation was used. ^*^p < 0.05; ^**^p < 0.01

### Mean differences in positive mental health, work stress, social support, emotion coping, situation coping, and resilience according to the demographic characteristics

Independent sample t-tests and one-way ANOVA were used to assess whether positive mental health, work stress, social support, emotional coping, situational coping, and resilience varied by gender, education level, and years of experience (Table 4). The findings revealed that female participants experienced higher levels of work stress than their male counterparts (M = 25.3, SD ± 4.80, *p* < 0.001). Regarding education, individuals with a BSN degree or higher reported greater social support (M = 3.49, SD ± 0.61, *p* = 0.008) and resilience (M = 3.20, SD ± 0.52, *p* = 0.001) compared to those with a diploma. Participants with less than 10 years of experience reported greater social support (M = 3.52, SD ± 0.66, *p* = 0.01), emotional coping (M = 3.35, SD ± 0.65, *p* = 0.014), and resilience (M = 3.39, SD ± 0.52, *p* = 0.006).

**Table 4:** Mean differences in participants’ positive mental Health, work Stress, social Support, emotion Coping, situation Coping, and resilience (*N* = 300)

		Positive Mental Health		Work Stress		Social Support		Emotion Coping		Situation Coping		Resilience	
		M (SD)	*p*	M (SD)	*p*	M (SD)	*p*	M (SD)	*p*	M (SD)	*p*	M (SD)	*p*
Gender	Male	2.8 (0.43)	0.38	23.4 (3.57)	< 0.001	3.3 (0.66)	0.158	3.18 (0.73)	0.08	3.17 (0.69)	0.21	3.2 (0.55)	0.09
	Female	2.8 (0.48)		25.3 (4.80)		3.4 (0.66)		3.29 (0.64)		3.24 (0.68)		3.3 (0.54)	
Level of Education	Diploma	2.78 (0.45)	0.07	24.8 (4.47)	0.46	3.3 (0.70)	0.008	3.2 (0.68)	0.09	3.17 (0.61)	0.09	3.2 (0.52)	0.01
	BSN or higher	2.9 (0.48)		24.7 (4.66)		3.49 (0.61)		3.3 (0.66)		3.2 (0.75)		3.3 (0.56)	
Length of Experience	< 10 years	2.87 (0.46)	0.16	24.28 (4.63)	0.18	3.52 (0.66)	0.011	3.35 (0.65)	0.014	3.31 (0.65)	0.09	3.39 (0.52)	0.006
	11–20 year	2.79 (0.47)		25.3 (4.02)		3.29 (0.69)		3.24 (0.66)		3.16 (0.73)		3.23 (0.56)	
	> 20 years	2.73 (0.48)		25.0 (5.3)		3.29 (0.56)		3.03 (0.67)		3.1 (0.67)		3.14 (0.52)	

Independent sample *t*-tests and one-way ANOVA were used

### The influence of positive mental health and work stress on resilience

Multiple linear regression was used to determine whether positive mental health and work stress predict resilience (Table 5). The regression model was significant [F (2, 297) = 49.69, adjusted R^2^ = 0.246, *p* < 0.001)]. Positive mental health (β = 0.535, *p* < 0.001) and work stress (β = 0.015, *p* = 0.015) were significant predictors of resilience, accounting for 24.6% of the variance.

**Table 5:** The influence of positive mental health and work stress on resilience (*N* = 300)

Model	Unstandardized Coefficients		Standardized Coefficients	t	*p*-value
	B	Std. Error	Beta		
(Constant)	2.15	0.246		8.76	< 0.001
Positive Mental Health	0.535	0.059	0.463	9.06	< 0.001
Work Stress	−0.015	0.006	−0.125	−2.44	0.015
Model Summary	F(2, 297) = 49.69, adjusted R^2^ = 0.246, *p* < 0.001				

^a^ Age = 20–22 years vs. 23–26 years. * *p* < 0.05, ** *p* < 0.001

## Discussion

This study investigated the association between positive mental health, work stress, and resilience among MHNs. 51% of the participants held a Bachelor’s degree in nursing, and the majority had less than ten years of professional experience in the nursing field. This finding aligns with a qualitative study in Saudi Arabia and an Australian cross-sectional study [1, 31]. This observation indicates that a nursing workforce, mainly consisting of individuals with bachelor’s degrees and less experience, showcases ongoing professional growth and rejuvenation in both Arabic and Western nations.

Regarding positive mental health, the results indicated that the participants had good positive mental health. Similarly, a study on nurses in acute care settings reported medium to high positive mental health levels [32]. In another study, healthcare workers (including nurses) reported that they had moderate mental health levels during the COVID-19 pandemic [33]. By contrast, a systematic review and meta-analysis revealed a decline in mental health among nurses during the pandemic [34]. The discrepancies between our findings and those of earlier studies may be attributed to the heightened challenges and responsibilities encountered during the various phases of the pandemic, as well as the employment of diverse mental assessment instruments. This observation underscores the necessity for organizational managers to consider the factors influencing nurses’ mental well-being when developing strategies aimed at enhancing their mental health.

Previous studies have reported moderate resilience levels among psychiatric nurses [24, 35, 36]. Supporting our findings, which revealed that more than two-thirds of the participants had moderate resilience levels, and no significant difference was found between males and females in terms of resilience level. However, some studies have reported conflicting results regarding resilience levels. These studies reported low to high resilience levels among mental health nurses, depending on the nurses’ coping strategies [37], whereas another study reported low resilience levels among nurses [38]. These discrepancies may be attributed to varying environmental factors within the workplace, shedding light on the organizational roles in identifying factors that affect the level of resilience among mental health nurses.

Our study found that most participants experienced moderate stress, notably that female stress levels were significantly higher than those of males, much of which was due to inadequate recognition and negative effects on their emotional and physical well-being. This finding aligns with an Ethiopian study revealing that approximately half of the enrolled nurses experienced moderate stress levels, which were caused by workload, death, rotating shifts, and social responsibilities [39]. In contrast, previous studies have revealed that nurses reported a higher level of stress when working in a public hospital compared to a private hospital, as well as in intensive care units [40, 41]. This difference could be attributed to the nature of the duties assigned in these units, which place nurses under more stress. Moreover, the use of different measurement tools may explain this contradiction. The comparative study employed the 34-item Nurse Stress Scale, which captures a broad range of stressors. In contrast, the present study utilized the shorter Work Stress Scale (WSS), consisting of only eight items, which may provide a more focused but less comprehensive assessment of stress.

Given that females reported higher stress levels despite comparable resilience in this study, it is crucial to implement policy-level measures, such as establishing safe nurse staffing standards and creating organizational recognition and support initiatives, to address these gender-based stress differences and enhance overall workforce well-being resources [42].

This study also determined the association between age, positive mental health, work stress, and resilience. Age was associated with work stress but not positive mental health and resilience. This indicates that different age groups experience stress differently, which is similar to a study that reported that younger nurses experienced higher stress levels than the older ones, while age was not correlated with mental health or coping strategies [40]. Other researchers reported higher levels of stress among nurses aged 30–34, with a significant association between age and stress [43]. By contrast, a recent Iranian study found that age was not correlated with occupational role stress among nurses [44].

The third aim of this study was to determine whether positive mental health, work stress, social support, emotional coping, situational coping, and resilience varied based on gender, education level, and years of experience. No significant difference in positive mental health was found between males and females. Similarly, a study on psychiatric nurses revealed that mental health did not differ between genders [45]. A multi-country study among healthcare workers, including nurses, reported no gender differences in mental health [46]. However, female participants comprised around two-thirds of the total sample, raising questions about the correlation between the study variables across different genders.

We found that males and females used similar emotional and situational coping strategies, consistent with a previous study that reported no significant differences between male and female nurses in their use of coping strategies [47]. By contrast, other studies have reported that female college students tend to use coping strategies more than their male counterparts [48, 49]. This may indicate that gender does not impact nurses’ coping strategies; however, there are differences among younger students. This highlights the importance of educational programs in enhancing the coping strategies of male students.

Regarding gender and social support, our study revealed that social support did not differ between males and females. Conversely, a study examining the mediating role of gender in the relationship between social support and quality of life in different populations reported that both genders had a better quality of life when they had strong social support. However, this effect was stronger in women, indicating differences between genders [50]. This difference could be explained by the fact that the number of male nurses was less than half that of female nurses in our study.

The current study found that males and females were similar in terms of situational coping, emotional coping, and resilience. This finding aligns with a study reporting that male and female university students showed similar resilience levels [51]. Another study found that women were generally more effective in their daily coping styles than men, and active stress coping was higher in men than in women, but these differences were not significant [52]. Policy makers and organizations should consider gender equality in providing accessible support, taking into consideration sub-group or contextual differences.

Our findings revealed that work stress and positive mental health significantly predict resilience among mental health nurses, explaining 24.6% of the variance. The remaining variance could be influenced by unmeasured factors, such as organizational support, personal coping strategies, or environmental stressors. This supports findings that moderate stress may enhance resilience through adaptive coping, while positive mental health buffers the adverse effects of stress. However, other studies suggest that high stress can overwhelm coping mechanisms, reducing resilience [10, 53]. These results underscore the importance of promoting positive mental health and effective stress management to enhance resilience among mental health nurses [10].

### Relevance for clinical practice

The results of this study provide valuable insights into mental health nursing practice by highlighting the importance of fostering resilience among mental health nurses. Given the emotionally demanding nature of psychiatric care, special attention should be paid to job-related factors that significantly affect their psychological well-being. Strategies such as reducing excessive workload, ensuring adequate staffing, and strengthening interprofessional communication can help create a supportive work environment. For mental health nurses, these measures are essential for enhancing emotional stability, improving therapeutic relationships with patients, increasing job satisfaction, and ultimately preventing compassion fatigue and burnout. By addressing these factors, healthcare organizations can better support the retention and effectiveness of mental health nurses, thereby improving the overall quality of psychiatric care.

### Limitations and recommendations

This study has several limitations. First, in the Saudi Arabian context, cultural norms and stigma surrounding mental health often influence the accuracy of self-reporting and the types of resilience strategies individuals employ. For example, public stigma discourages help-seeking and may lead individuals to underreport psychological distress [54]. Recognizing these cultural dynamics is essential for interpreting our findings and for designing interventions. Future research should therefore move beyond descriptive approaches to include intervention-focused studies, such as trials testing resilience-building programs tailored to the Saudi cultural context.

Second, the data were collected at a single time point using convenience sampling, which may limit the generalizability of the findings to other settings. Future studies should use a longitudinal design to measure changes over time. Additionally, exploring diverse sampling methods, including random sampling, could enhance the robustness of future research findings. Incorporating qualitative data could also enrich the understanding of participants’ experiences and the contextual factors influencing their responses.

Third, self-report measures may enhance social desirability. Therefore, the findings should be interpreted with caution. Other researchers may use direct measures (i.e., cortisol level) to objectively assess participants’ stress levels. This approach could provide a more accurate depiction of stress responses and reduce biases associated with self-reporting. By addressing these limitations, future research can contribute more to the field and improve the applicability of the findings.

## Conclusion

This study highlights the critical link between positive mental health, work stress, and resilience among mental health nurses. Our findings show that positive mental health significantly enhances resilience, while stress, particularly among female nurses, diminishes it. These results emphasize the need to promote positive mental health and effective stress management. Despite varied findings in previous research, our study consistently highlights the importance of these factors for resilience in demanding professional settings. Effective positive mental health strategies and interventions must be implemented to alleviate work-related stress and improve nurses’ performance. Creating supportive work environments that foster collaboration and empower nurses to deliver high-quality care while reducing stressors is also crucial. Future research should explore factors contributing to these disparities and the long-term effects of stress on resilience. Ultimately, systemic reforms are essential to create supportive work environments that prioritize the mental well-being of healthcare professionals, fostering a more resilient nursing workforce and improving patient care.

## Supplementary Information

Supplementary Material 1.

## Data Availability

The datasets are available from the corresponding author [AA] upon reasonable request.
