# Supplementary material for: The influence of positive mental health and work stress on resilience among mental health nurses: a cross-sectional study
Source: BMC Med Educ. 2025 Dec 17;25:1698. doi: 10.1186/s12909-025-08249-6 (PMC12709729; doi:10.1186/s12909-025-08249-6)
Supplement: Supplementary file 1 — Supplementary Material 1. [file 12909_2025_8249_MOESM1_ESM.docx]

**The Influence of Positive Mental Health and Work Stress on Resilience Among Mental Health Nurses: A Cross-Sectional Study**

Authors: Adnan Innab^1^, Atallah Alenezi*^2^, Sahar Al-Ghareeb^3^, Mona Mostafa^4^

^1^ Associate Professor, Nursing Administration and Education Department,
College of Nursing, King Saud University, Riyadh 12372, Saudi Arabia
ainnab@ksu.edu.sa
ORCID: 0000-0002-9527-1078

^2^ Professor, College of Nursing,

Imam Mohammad Ibn Saud Islamic University (IMSIU), Riyadh, Saudi Arabia

akhalenezi@imamu.edu.sa

ORCID: 0000-0002-6272-5379

^3^ Lecturer, Fundamentals of Nursing Department, College of Nursing, Imam Abdulrahman Bin Faisal University, , Dammam, Saudi Arabia

Saalghareb@iau.edu.sa

ORCID: 0009-0007-6844-4539

^4^ Assistant Professor, Department of Nursing Sciences, College of Applied Medical Sciences, Shaqra University, Shaqra, Reyad, Saudi Arabia.

Assistant Professor, Psychiatric & Mental Health Nursing Department, Faculty of Nursing, Cairo University, Cairo, Egypt.

Email: [m.mohammad@su.edu.sa](mailto:m.mohammad@su.edu.sa)

ORCID: [0000-0002-8782-1295](https://orcid.org/0000-0002-8782-1295)

Phone #: +966535279865

Corresponding Author

^2^ Atallah Alenezi *

**Ethics approval and consent to participate**

Approval was obtained from Shaqra University’s Institutional Review Board (IRB) under reference number [ERC-SU-S-202400047]. All participants provide informed consent to take part in this research. This study adhered to the principles of the Declaration of Helsinki

**Competing interests**

The authors declare that they have no competing interests.

**Funding**

This work was supported and funded by the Deanship of Scientific at Imam Mohammad Ibn Saud Islamic University (IMSIU) (grant number: IMSIU-DDRSP2501).

**Data availability**

The datasets are available from the corresponding author [AA] upon reasonable request.

**Authors’ Contributions**

All the authors contributed to this study based on the criteria of the International Committee of Medical Journal Editors. All authors have approved the manuscript and agreed to submit it to the journal. Conceptualization, methodology & data collection (A.A., A.I., M.M.), writing-original draft preparation, review and editing (A.A., A.I., SG., M.M), The final manuscript was read, reviewed, and approved by all authors (A.A., A.I., SG., M.M).
